# Transcriptional Repression of CYP3A4 by Increased miR-200a-3p and miR-150-5p Promotes Steatosis *in vitro*

**DOI:** 10.3389/fgene.2019.00484

**Published:** 2019-05-28

**Authors:** Zhijun Huang, Mengyao Wang, Li Liu, Jinfu Peng, Chengxian Guo, Xiaoping Chen, Lu Huang, Jieqiong Tan, Guoping Yang

**Affiliations:** ^1^Center for Clinical Pharmacology, The Third Xiangya Hospital, Central South University, Changsha, China; ^2^Department of Pharmacy, The Third Xiangya Hospital, Central South University, Changsha, China; ^3^Xiangya School of Pharmaceutical Sciences, Central South University, Changsha, China; ^4^Institute of Clinical Pharmacology, Central South University, Changsha, China; ^5^Center for Medical Genetics, Life Science School, Central South University, Changsha, China

**Keywords:** non-alcoholic fatty liver disease, CYP3A4, miR-200a-3p, miR-150-5p, LO2 cell line

## Abstract

Hepatic cytochrome P450 enzyme activities correlate with non-alcoholic fatty liver disease (NAFLD) and hepatic steatosis. The decreased activity of CYP3A4, an important drug-metabolizing enzyme, is associated with the progression of NAFLD. CYP3A4 is predicted as a target gene of miR-200a-3p and miR-150-5p by MicroInspector and TargetScan algorithms analyses. Here, we found decreased CYP3A4 and increased miR-200a-3p and miR-150-5p in LO2 cells with free fatty acid (FFA)-induced steatosis. Dual-luciferase assay confirmed that both miR-200a-3p and miR-150-5p targeted the 3′-untranslated region (3′-UTR) of CYP3A4 and that such interaction was abolished by miRNA binding site mutations in 3′-UTR of CYP3A4. Using miR-200a-3p and miR-150-5p mimics and inhibitors, we further confirmed that endogenous CYP3A4 was regulated posttranscriptionally by miR-200a-3p or miR-150-5p. Moreover, miR-200a-3p and miR-150-5p inhibitors attenuated FFA-induced steatosis in LO2 cells, and such effect was dependent on CYP3Y4 expression. These results suggest that miR-200a-3p and miR-150-5p, through directly targeting 3′-UTR of CYP3A4, contribute to the development of FFA-induced steatosis.

## Introduction

Non-alcoholic fatty liver disease (NAFLD) has been the most common chronic liver disease in the world for the past 30 years (Younossi et al., 2018) with an estimated prevalence of 10–40% (Younossi et al., 2016). NAFLD includes a spectrum of disease from simple liver steatosis to non-alcoholic steatohepatitis (NASH). NASH is considered as a leading indication for liver transplants in the near future, as it increases the risk of hepatocarcinoma (HCC) (Sattar et al., 2014; Diehl and Day, 2017).

Several genetic defects and environmental factors have been implicated in the pathogenesis of NAFLD. Specifically, a few studies have reported that NAFLD is associated with decreased expression and function of CYP3A4, a major member of the hepatic cytochrome P450 superfamily contributing to the metabolism of 45–60% of all drugs used in the clinical setting (Wojnowski and Kamdem, 2006; Anglicheau et al., 2007). Donato et al. (2006) observed a significantly reduced CYP3A4 activity in both human liver tissue with steatosis and fat-overloaded hepatocytes cultured *in vitro*. Similar results were shown in nutritionally obese mice (Yoshinari et al., 2006; Maximos et al., 2017). Compared with healthy controls, NAFLD patients showed higher plasma concentration of CYP3A4 substrate, indicating impaired CYP3A4 function in NAFLD patients (Woolsey et al., 2015). Decreased protein expression and activity of CYP3A4 were observed with NAFLD development (Fisher et al., 2009). A recent study further confirmed reduced protein level and activity of CYP3A4 in liver tissues of NAFLD patients as compared to those of controls (Jamwal et al., 2018). However, despite all these observations, the underlying mechanism regulating the expression and function of CYP3A4 in NAFLD remains unclear.

MicroRNAs (miRNAs/miRs) are small non-coding RNAs that negatively regulate gene expression through binding to the 3′-untranslated region (3′-UTR) of mRNAs, thus altering the expression and function of various genes, including CYP3A4 (Pan et al., 2009; Swathy et al., 2017; Yan et al., 2017). miRNA-34a, miRNA-122, and miRNA-192 are considered as potential biomarkers of NAFLD staging (Liu et al., 2018). Importantly, high-throughput sequencing revealed that expressions of miR-150-5p and miR-200a-3p were significantly higher in NAFLD with fibrosis than in NAFLD without fibrosis (Leti et al., 2015). Therefore, we hypothesize that significant change of hepatic miRNAs in NAFLD could regulate CYP3A4 expression posttranscriptionally. Here, we report that miR-150-5p and miR-200a-3p directly regulate CYP3A4 and are involved in free fatty acid (FFA)-induced steatosis.

## Materials and Methods

### Reagents

Sodium salts of palmitic acid (PA) (P9767) and oleic acid (OA) (O7501), fatty acid (FA) free bovine serum albumin (BSA) (A8806), and BODIPY 493/503 (790389) were purchased from Sigma-Aldrich (MO, United States), and RPMI 1640 (11875-093) medium and fetal bovine serum (FBS) (26140079) were from GIBCO (Invitrogen, CA, United States). Lipofectamine^TM^ 2000 (11668019) was from Invitrogen (CA, United States). Oligonucleotide primers for CYP3A4 were synthesized by Sangon Biotech (Shanghai, China). All miRNA mimics, miRNA mimic negative controls, miRNA inhibitors, miRNA inhibitor negative controls, and primers for miRNA RT-qPCR were purchased from RiboBio (Guangzhou, China). All other chemicals and solvents were of the highest commercial grades.

### Cell Culture Model of Hepatic Steatosis *in Vitro*

LO2 cells were provided by Stem Cell Bank, Chinese Academy of Sciences and cultured in RPMI 1640 medium supplemented with 10% FBS at 37° C/5% CO_2_. Steatosis was induced as previously described (Feldstein et al., 2004; Ricchi et al., 2009). PA and OA were codissolved in 10% FA-free BSA prepared in H_2_O. In accordance with previous studies (Wang et al., 2014), LO2 cells were exposed to a mixture of 1 mM OA and PA (final ratio 2:1) for 24 h. After 24 h of incubation, lipid droplets were stained by BODIPY according to a previously reported protocol (Qiu and Simon, 2016).

### *In silico* Identification of Putative miRNA Binding Sites

The 3′-UTR sequences of human CYP3A4 (GenBank sequence NM_017460) were searched for the antisense matches to individual miRNAs using MicroInspector (Rusinov et al., 2005) and Target Scan (Lewis et al., 2005).

### Transfection

All transfections were performed by Lipofectamine^TM^ 2000 according to the manufacturer’s instructions.

### Real-Time PCR

Small RNA was extracted with an E.Z.N.A miRNA kit (Omega BIO-TEK, GA, United States) and reverse transcribed using PrimeScript RT Reagent Kit (Roche, Basel, Switzerland) with primers for specified miRNAs. The oligonucleotide sequences of STEM-LOOP RT primers were designed (Kramer, 2011) as follows: miR-200a-3p, 5′-GTCGTATCCAGTGCAGGGTCCGAGGTATTCGCACTGGATACGACacatcgtt-3′; miR-150-5p, 5′-GTCGTATCCAGTGCAGGGTCCGAGGTATTCGCACTGGATACGACctgtcccc-3′; U6, 5′-GTCGTATCCAGTGCAGGGTCCGAGGTATTCGCACTGGATACGACaaaaatat-3′. Real-time PCR was performed with SYBR Green PCR Master Mix (Roche, Basel, Switzerland) using the following conditions: 95° C for 10 min followed by 40 cycles of amplification at 95° C for 10 s and 59° C for 30 s. Mature miR-200a-3p and miR-150-5p levels were normalized with U6. The oligonucleotide sequences of qPCR primers were as follows: miR-200a-3p, 5′-CACGCAtaacactgtctggtaa-3′; miR-150-5p, 5′-CACGCActggtacagggcctgg-3′; U6, 5′-CACGCAgcaaggatgacacgcaa-3′; general reverse primer, 5′-CACGCATGGAAGGACGGG-3′.

To inhibit or induce miR-150-5p or miR-200a-3p, transient transfection of miRNA inhibitors or mimics (100 nM) was performed in LO2 cells using Lipofectamine^TM^ 2000. Specific miRNA-150 or miRNA-200a inhibitors or mimics were commercially purchased from RiboBio (Guangzhou, China), including anti-miRNA-150 (target sequence 5′-CUGGUACAGGCCUGGGGGACAG-3′), anti-miRNA-200a (target sequence 5′-UAACACUGUCUGGUAACGAUGU-3′), syn-miRNA-150 (target sequence 5′-CUGGUACAGGCCUGGGGGACAG-3′), and syn-miRNA-200a (target sequence 5′-UAACACUGUCUGGUAACGAUGU-3′). miScript inhibitor negative control (100 nM) (RiboBio, Guangzhou, China) was used as internal reference for normalization.

Twenty-four hours after transfection, cells were treated with a mixture of 1 mM OA and PA (final ratio 2:1) for 24 h. To confirm the effect of miR-150-5p or miR-200a-3p inhibition or induction on CYP3A4 mRNA, total RNAs were extracted from LO2 cells with TRIzol (Life Technologies, CA, United States). Total mRNAs were reverse transcribed into cDNAs by the PrimeScript RT Reagent kit. Real-time PCR was performed by the SYBR Green PCR Master Mix using the following conditions: 95° C for 10 min followed by 40 cycles of amplification at 95° C for 10 s and 59° C for 30 s. GAPDH (forward primer: AGAAGGCTGGGGCTCATTTG; reverse primer: AGGGGCCATCCACAGTCTTC) was used as an internal control to normalize CYP3A4 expression (forward primer: CCCGTTGTTCTAAAGGTTGA; reverse primer: TCTGGTGTTCTCAGGCACAG). qPCR was quantified using the formula 2^−ΔΔCT^ and plotted as x-fold to the control.

### Western Blot

Cells in six-well plates were harvested posttransfection and treatment, and whole-cell lysates were prepared with RIPA lysis buffer (Beyotime, Beijing, China) supplemented with complete protease inhibitor and phenylmethanesulfonyl fluoride (Beyotime, Beijing, China). Protein concentrations were determined with the BCA Protein Assay Kit (Beyotime, Beijing, China). Whole-cell protein (20 μg) was separated on SDS-PAGE and electrophoretically transferred onto PVDF membrane (Millipore, CA, United States). The membrane was incubated with a selective rabbit anti-human CYP3A4 polyclonal antibody (Millipore, CA, United States) or mouse anti-human GAPDH antibodies (Zhongshan Inc., Guangzhou, China), and subsequently with the secondary antibody of HRP goat anti-rabbit IgG (Zhongshan Inc., Guangzhou, China) or rabbit anti-mouse IgG (Zhongshan Inc., Guangzhou, China). Images were acquired with GE Healthcare ImageQuant 350, and band densities were quantified with GeneTools (SynGene, Cambridge, United Kingdom).

### Construction of Reporter Plasmids

The 3′-UTR of CYP3A4 gene corresponding to 1,620–2,792 nt (1,173 bp; accession no. NM_001202855) was cloned into pmiR-RB-REPORT^TM^ vector *via XhoI* and *NotI* restriction sites. The primers used for construction of wild-type CYP3A4 3′-UTR reporter plasmid were as follows: h-CYP3A4-F, 5′-CTTGACTCGAGATTTTCCTAAGGACTTCTGC-3′; h-CYP3A4-R, 5′-ATTGCGGCCGCAGGCTTATTGCTCAATC-3′. The sequence of the recombinant clones was confirmed by DNA sequencing and named as pmiR-RB-REPORT^TM^ CYP3A4 3′-UTR WT. miRNA-200a binding site mutant (AGTGTTA changed to TGTGCCA) and miRNA-150 binding site double mutant (TTCCCAG changed to ATCCGAT and TGGGAGA changed to AGGCATA) were constructed using Q5^®^ Site-Directed Mutagenesis Kit (NEB, MA, United States) and confirmed by DNA sequencing. The firefly luciferase gene used 3′-UTR of CYP3A4 as the report luciferase, with Renilla luciferase gene as an internal control.

### Dual-Luciferase Assay

The LO2 cells were seeded into 24-well plates. Firefly luciferase (0.1 μg) containing 3′-UTR of CYP3A4 in pmiR-RB-REPORT^TM^ vector, along with miR-200a-3p or miR-150-5p mimic, was transfected into LO2 cells with Lipofectamine 2000. After 24 h of incubation, luciferase activities were measured with a luminometer (Tecan Infinite 200 Pro, Switzerland) using the Dual-Luciferase Reporter Assay System (Promega, Valencia, CA, United States). Firefly luciferase activity was normalized by Renilla luciferase activity and compared between different treatments.

### Statistical Analysis

All values were expressed as the mean ± SEM. Comparisons of variables between groups were performed with an unpaired two-tailed Student’s *t*-test. *P* < 0.05 was considered statistically significant.

## Results

### CYP3A4 Expression Is Decreased in FFA-Induced Steatosis Cells

**FIGURE 1:**
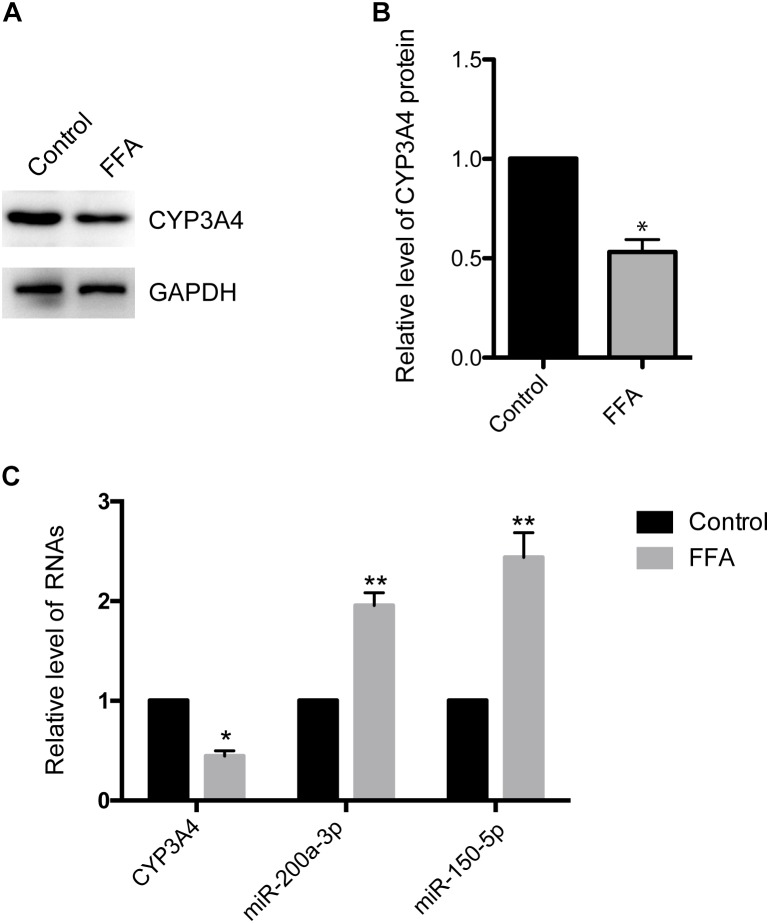
Free fatty acid (FFA) induces decrease of CYP3A4 and increase of miR-200a-3p and miR-150-5p. **(A)** The CYP3A4 protein level was determined by Western blot and normalized with GAPDH. The control was treated solely with 1% BSA, while steatosis cells were exposed to 1 mM FFA (a mixture of OA and PA with final ratio 2:1) for 24 h. **(B)** Quantitative analysis of CYP3A4 protein shown in **(A)**. **(C)** CYP3A4 mRNA level was determined by real-time PCR and normalized with GAPDH. Mature miR-200a-3p and miR-150-5p levels in steatosis cells were determined by real-time PCR and normalized with U6 snRNA. Values were expressed as mean ± SEM for three independent experiments. ^∗^*p <* 0.05, ^∗∗^*p <* 0.01, versus control.

CYP3A4 activity is reduced in human NAFLD as well as in mouse and *in vitro* cell models of the disease (Kolwankar et al., 2007; Woolsey et al., 2015). Here, we investigated CYP3A4 expression in FFA-induced steatosis cells *in vitro*. As shown in Figure 1A,B, CYP3A4 protein level was decreased in FFA-induced steatosis cells compared to control. Similarly, a significant decrease of CYP3A4 mRNA was observed in FFA-induced steatosis cells compared to control (Figure 1C).

### Increased Mature miR-200a-3p and miR-150-5p in FFA-Induced Steatosis Cells

**FIGURE 2:**
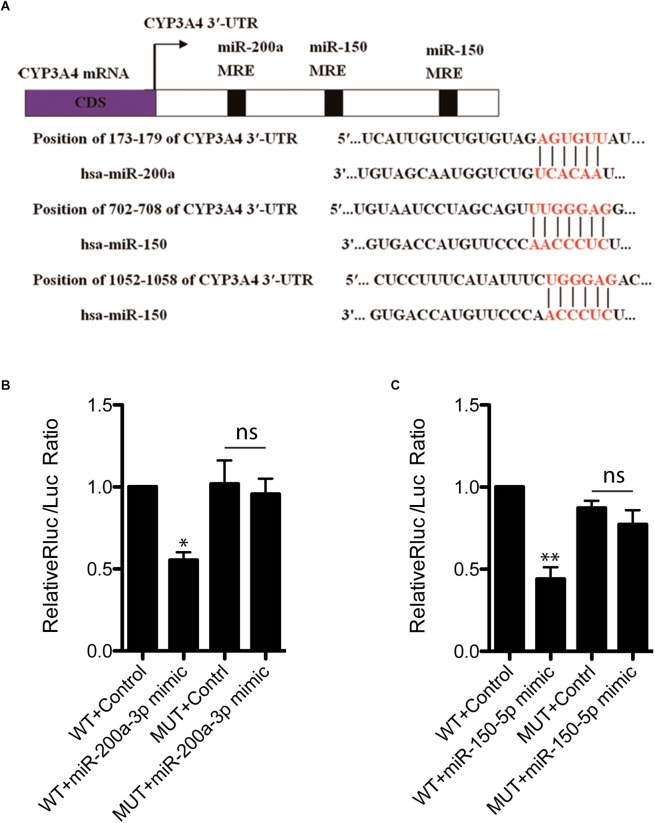
miR-200a-3p and miR-150-5p mimics down-regulate CYP3A4 expression by binding to its 3′-UTR. **(A)** The miR-200a-3p and miR-150-5p MRE sites (miRNA recognition element) within human CYP3A4 3′-UTR predicted by MicroInspector and/or TargetScan are both conserved. **(B)** Evaluation of binding ability of miR-200a-3p **(B)** or miR-150-5p **(C)** mimics to CYP3A4 3′-UTR-Luc WT and miRNA binding site mutants (MUT). LO2 cells were cotransfected with a firefly luciferase reporter vector containing the indicated CYP3A4 3′-UTR constructs, a Renilla luciferase reporter as internal control, and treated with miR-200a-3p **(B)** or miR-150-5p **(C)** mimics. Values were mean ± SEM for three independent experiments. ^∗^*p <* 0.05, ^∗∗^*p <* 0.01, versus control; ns, not significant.

To investigate whether decreased CYP3A4 in FFA-induced steatosis cells was due to miRNAs regulation, we used MicroInspector and TargetScan algorithms to screen antisense matches of CYP3A4 3′-UTR against human miRNAs. CYP3A4 was predicted to be a target gene of miR-200a-3p and miR-150-5p (Figure 2A). Next, we determined the expression levels of mature miR-200a-3p and miR-150-5p in FFA-induced steatosis cells and found that both mature miR-200a-3p and miR-150-5p were higher in FFA-induced steatosis cells than in control cells (Figure 1C).

### miR-200a-3p and miR-150-5p Regulate CYP3A4 Expression by Targeting Its 3′-UTR

To investigate whether CYP3A4 can be directly regulated by mature miR-200a-3p and miR-150-5p, CYP3A4 3′-UTR WT and the two miRNA binding site mutants were cloned separately into pmiR-RB-REPORT^TM^ vector for dual-luciferase assay. We showed that both miR-200a-3p and miR-150-5p interacted with CYP3A4 3′-UTR WT, but not the two miRNA binding site mutants (Figure 2B,C).

### miR-200a-3p and miR-150-5p Down-Regulate CYP3A4 Expression in FFA-Induced Steatosis Cells

**FIGURE 3:**
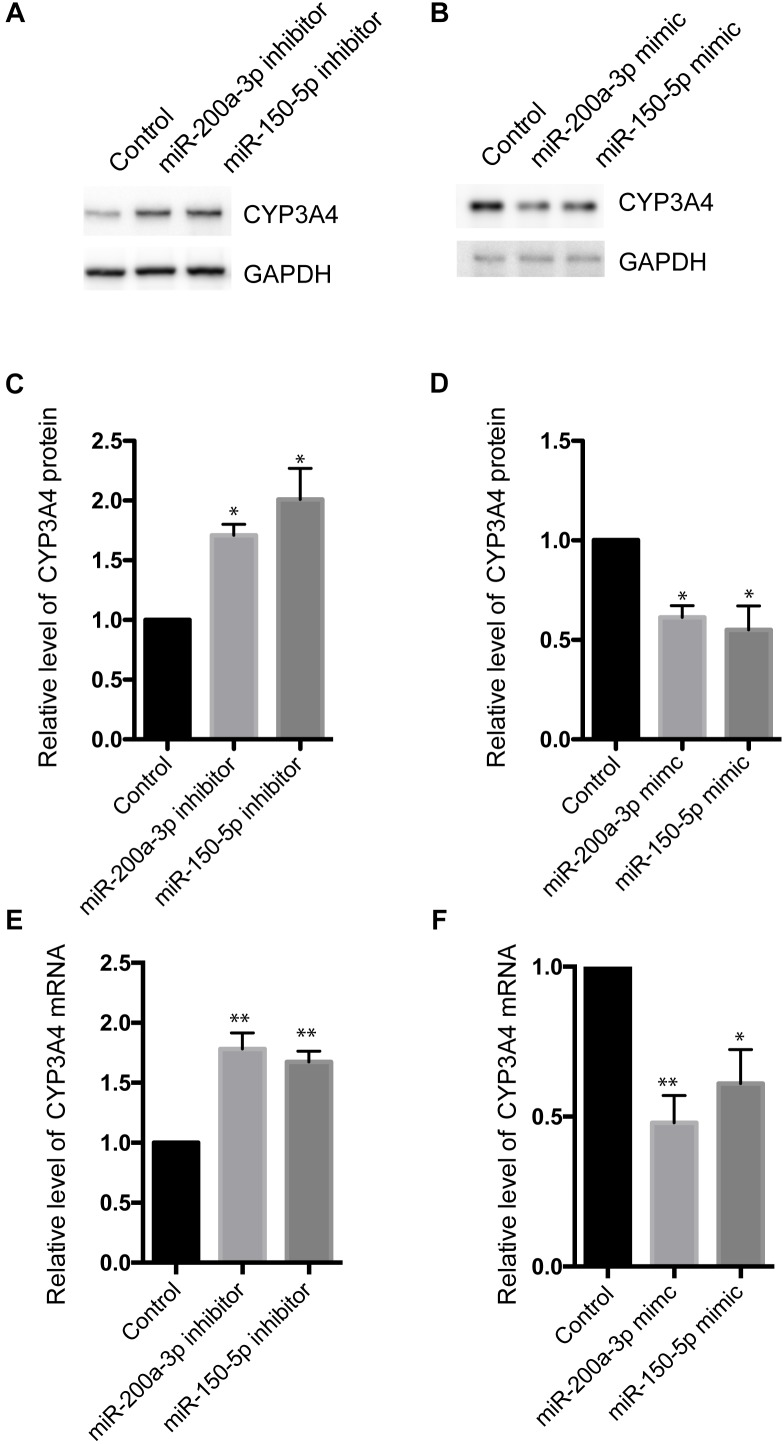
Effects of miR-200a-3p and miR-150-5p mimics or inhibitors on mRNA and protein levels of CYP3A4 in LO2 cells. **(A,B)** miR-200a-3p and miR-150-5p inhibitors **(A)** or mimics **(B)** were transfected into LO2 cells. After 24 h, the cells were harvested. CYP3A4 protein level was determined by Western blot and normalized with GAPDH. **(C,D)** Quantitative analysis of CYP3A4 shown in **(A,B)**, respectively. **(E,F)** The mRNA level of CYP3A4 was determined by real-time PCR and normalized with GAPDH in cells transfected with miR-200a-3p or miR-150-5p inhibitors **(E)** or mimics **(F)**. Values were mean ± SEM for three independent experiments. ^∗^*p <* 0.05, ^∗∗^*p*<0.01, versus control.

To investigate the effect of miR-200a-3p and miR-150-5p on CYP3A4 expression, we examined changes of CYP3A4 mRNA and protein levels in response to inhibition or induction of miR-200a-3p or miR-150-5p. miR-200a-3p or miR-150-5p inhibitor increased CYP3A4 protein (Figure 3A–D) and mRNA (Figure 3E) levels. In addition, CYP3A4 protein and mRNA (Figure 3F) levels were decreased after miR-200a-3p or miR-150-5p mimic treatment.

### miR-200a-3p or miR-150-5p Inhibitor Regulates FFA-Induced Steatosis *via* CYP3A4

**FIGURE 4:**
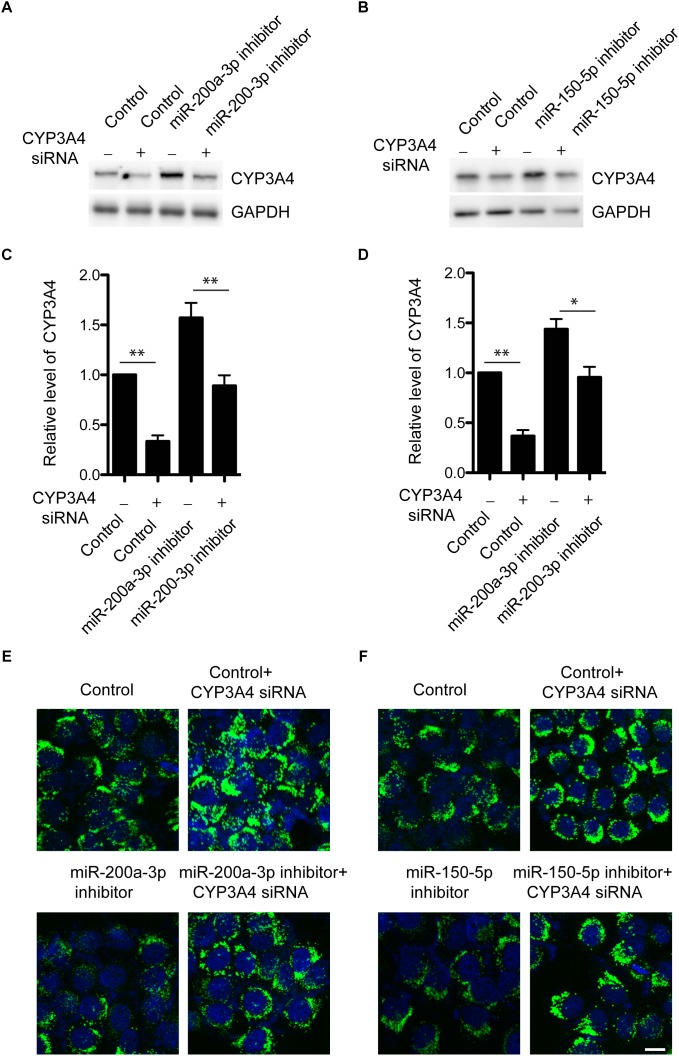
Knockdown of CYP3A4 abolishes reduced FFA-induced steatosis mediated by miR-200a-3p and miR-150-5p inhibitors. **(A,B)** miR-200a-3p **(A)** and miR-150-5p **(B)** inhibitors were transfected into LO2 cells with or without knockdown of CYP3A4. After 24 h, cells were harvested. CYP3A4 protein level was determined by Western blot and normalized with GAPDH. **(C,D)** Quantitative analysis of CYP3A4 protein shown in **(A,B)**, respectively. **(E,F)** miR-200a-3p and miR-150-5p inhibitors were transfected into LO2 cells with or without knockdown of CYP3A4. After 24 h, cells were exposed to 1 mM FFA or vehicle. The BODIPY 493/503 staining was performed to assess cellular steatosis. Green fluorescence indicated lipid droplets. The cell nucleus was stained by DAPI (Blue). ^∗^*p <* 0.05, ^∗∗^*p <* 0.01, bar = 10 μm.

We have demonstrated that miR-200a-3p or miR-150-5p negatively regulated CYP3A4 expression. Because of the importance of CYP3A4 activity in steatosis development (Donato et al., 2006; Hu et al., 2014), we then determined whether CYP3A4 mediated the regulatory effect of miR-200a-3p or miR-150-5p inhibitor on FFA-induced steatosis. miR-200a-3p or miR-150-5p inhibitor increased CYP3A4 expression, which was abolished by CYP3A4 knockdown (Figure 4A–D). More importantly, miR-200a-3p or miR-150-5p inhibitor reduced FFA-induced steatosis assessed by the BODIPY 493/503 staining, and this effect was abrogated by CYP3A4 gene silencing (Figure 4E,F). This suggested that miR-200a-3p or miR-150-5p inhibitor, through up-regulating CYP3A4, protected against FFA-induced steatosis.

## Discussion

In recent years, NAFLD has emerged as a major public health concern characterized by elevated serum FFAs and hepatocyte lipoapoptosis (Feldstein et al., 2003). Several reports have indicated the importance of both quantitative and qualitative (e.g., saturated versus unsaturated FAs) changes in dietary FAs as underlying causative mechanisms for NAFLD in both rodent models and humans (Nehra et al., 2001; Musso et al., 2003; Wang et al., 2006). Increased supply of FAs or decreased lipid clearance in hepatocytes can set off esterification of FAs and formation of lipid droplets (Anderson and Borlak, 2008). In terms of mechanism, enzymes, important regulators of plasma and tissue FA composition, signaling pathways, and transcriptional factors controlling FA synthesis and gluconeogenesis have all been implicated in NAFLD (Malhi and Gores, 2008; Mota et al., 2016). Here, we demonstrated that miR-200a-3p and miR-150-5p, through directly targeting 3′-UTR of CYP3A4, contributed to the development of FFA-induced steatosis *in vitro*.

Previous studies have evaluated the relationship between NAFLD and hepatic CYP3A activity. Fisher et al. (2009) explored the relationship between hepatic CYP3A4 and non-alcoholic hepatic steatosis in human liver samples. It was found that CYP3A4 protein expression and activity decreased with the progression of NAFLD. Other studies (Kolwankar et al., 2007; Jamwal et al., 2018) showed that liver samples with steatosis had significantly lower hepatic CYP3A4 activity than those without steatosis, which was consistent with *in vitro* findings in long-chain FFA-induced steatosis. CYP3A4 mRNA and protein also decreased significantly in fatty mice induced by a high-fat diet (Yoshinari et al., 2006).

Although most of the CYPs are found within endoplasmic reticulum, CYP3A4 localizes in mitochondria and synthesizes arachidonic acid (AA)-derived epoxyeicosatrienoic acids (EETs), which promotes electron transport chain/respiration and mitochondrial function (Guo et al., 2017). It is well known that CYP enzyme activities are affected by steatosis, which poses major impact on drug metabolism and drug-induced hepatotoxicity (Gomez-Lechon et al., 2009). However, our study suggests that increased CYP3A4 expression prevents LO2 cells from FFA-induced steatosis. Our results demonstrate that CYP3A4 activity impairment is not only induced by hepatic steatosis but also a factor further promoting hepatic steatosis. This could explain the phenotype of cyp3a-null male mice, including increases in weight, liver triglycerides, and total lipids (Kumar et al., 2018).

It was previously shown that CYP3A4 could be regulated by miRNAs *via* direct and indirect targeting (Takagi et al., 2008; Pan et al., 2009; Swathy et al., 2017; Yan et al., 2017). Hepatic miRNA changes in NAFLD have been confirmed in several studies of dietary NASH in mice and rats (Pogribny et al., 2010; Alisi et al., 2011), as well as serum and liver tissue of NAFLD patients (Leti et al., 2015; Liu et al., 2018). In this study, we reported two new CYP3A4 regulators, including miR-200a-3p and miR-150-5p, in FFA-induced steatosis. Our results showed that expressions of miR-200a-3p and miR-150-5p increased significantly in hepatocytes with steatosis as compared to normal hepatocytes, which is consistent with previous studies reporting an increase of miR-200a-3p in NAFLD (Cheung et al., 2008; Alisi et al., 2011). More importantly, miR-200a-3p or miR-150-5p inhibitor can alleviate FFA-induced steatosis. Thus, our findings provide a potential therapeutic target for the use of miR-200a-3p and miR-150-5p inhibitors to protect against steatosis development.

## Author Contributions

ZH and MW performed the experiments and wrote the manuscript. LL, JP, CG, XC, LH, and JT performed the experiments. ZH and GY designed the project and provided supervision and support for the project.

## Conflict of Interest Statement

The authors declare that the research was conducted in the absence of any commercial or financial relationships that could be construed as a potential conflict of interest.
